# Alcohol in the Army

**Published:** 1899-05

**Authors:** 


### Alcohol in the Army.

The British authorities some time ago made a test of the alleged value of alcohol when men are subjected to unusual and exhausting labor. Experiments were made at different times and under varying conditions with three regiments from each of several brigades. In one every man was forbidden to drink any alcohol whatever while the test lasted; in the second malt liquor only was taken; in the third a ration of whisky was given to each man. The whisky drinkers manifest more dash at first, but generally in about four days showed signs of weakness and fatigue; those given malt liquor displayed less dash at first, but their endurance lasted somewhat longer, while the abstainers improved daily in alertness and staying powers. In the German army experiments are being made with sugar, which is claimed to have such great sustaining powers that it is proposed to serve it as an extra ration when unusual fatigues are to be borne.—*Medical Record.*

